# Supplementary material for: Cohort profile: The Swedish Tattoo and Body Modifications Cohort (TABOO)
Source: BMJ Open. 2023 May 4;13(5):e069664. doi: 10.1136/bmjopen-2022-069664 (PMC10163470; doi:10.1136/bmjopen-2022-069664)
Supplement: Supplementary data [file bmjopen-2022-069664supp001.pdf]

Cohort Profile: The Swedish Tattoo and Body Modifications Cohort (TABOO)

Supplementary file

Table S1. Detailed description of exposure data and personal characteristics collected through the TABOO questionnaire.

Variable	Who got the question	Response options	Multiple responses possible	Comment
Tattoos				
Tattoo status	All	Yes (including any removed tattoo)<br>No		
Type of tattoo	Tattoo status = yes	Decorative<br>Cosmetic<br>Medical	x	
Age at first tattoo	Tattoo status = yes	Years		
Age at last tattoo	Tattoo status = yes	Years		
Tattooed anatomical sites	Tattoo status = yes	1: Head<br>2: Neck<br>3: Right upper arm, chest, upper back<br>4: Left upper arm, chest, upper back<br>5: Right lower arm, hand<br>6: Left lower arm, hand<br>7: Abdomen, groin, genitals, lower back, buttocks<br>8: Right thigh, knee<br>9: Left thigh, knee<br>10: Right lower leg, foot<br>11: Left lower leg, foot	x	The numbered response options refer to anatomical sites in Figure 1, which was included in the questionnaire
Area of tattooed body surface	Tattoo status = yes	<1 hand<br>1 to 5 hands<br>>5 hands		
By whom and where tattoo was made	Tattoo status = yes	Professional tattoo artist in tattoo studio<br>Professional tattoo artist at other facility<br>Cosmetic tattoo artist in cosmetic tattoo studio, beauty salon, or plastic surgery clinic<br>Health care professional at hospital or other health care establishment	x	

Nielsen C, *et al. BMJ Open* 2023; 13:e069664. doi: 10.1136/bmjopen-2022-069664

Cohort Profile: The Swedish Tattoo and Body Modifications Cohort (TABOO)

		Other irrespective of where		
Number of tattoos	Tattoo status = yes	1<br>2 to 3<br>4 to 5<br>6 to 9<br>≥10		Tattoos considered separate if ≥20 cm between them<br>Cover-ups counted as 2 tattoos
Number of tattoo sessions	Tattoo status = yes	1<br>2 to 3<br>4 to 5<br>6 to 9<br>≥10		
Tattooed under the influence of alcohol or drugs	Tattoo status = yes	Yes<br>No		
Tattoo colours	Tattoo status = yes	Black<br>Gray<br>Brown<br>red<br>Blue<br>Green<br>Yellow<br>White<br>Purple<br>Pink<br>Orange<br>Turquoise<br>Skin tone (injected colour)<br>Other	x	
Geographical regions of tattooing	Tattoo status = yes	Sweden<br>Nordic countries (not Sweden)<br>Rest of Europe (not Nordic countries)<br>Asia<br>Oceania<br>USA<br>Other	x	
Other body modifications				
Piercing status	All	Yes<br>No, had earlier<br>No, never		Including earlobes
Scarification status	All	Yes<br>No		Scars obtained from branding or cutting
Henna tattoo status	All	Yes, black<br>Yes, red<br>No	x	Tattoo painted on skin
Laser treatment: Tattoo removal	Tattoo status = yes	Yes<br>No		
Laser treatment: Hair removal	All	Yes<br>No		Intense pulsed light (IPL given as an example)
Laser treatment: Mole or hyperpigmentation removal	All	Yes<br>No		Intense pulsed light (IPL given as an example)

Cohort Profile: The Swedish Tattoo and Body Modifications Cohort (TABOO)

Laser treatment: Thread vein removal	All	Yes<br>No		Intense pulsed light (IPL given as an example)
Laser treatment: Facial wrinkles reduction	All	Yes<br>No		Intense pulsed light (IPL given as an example)
Laser treatment: Other reason	All	Yes<br>No		Intense pulsed light (IPL given as an example)
Anatomical site of treatment	Laser treatment (specified) = yes	Face<br>Arms<br>Legs<br>Back<br>Chest and/or abdomen<br>Other	x	Repeated for each of the six reasons for laser treatment
Where laser treatment was performed	Laser treatment (specified) = yes	Beauty salon, beauty clinic, or plastic surgery clinic<br>Health care establishment<br>Home<br>Other<br>(For tattoo removal, the option tattoo artist was provided)		Repeated for each of the six reasons for laser treatment
Number of laser treatments	Laser treatment (specified) = yes	1 to 2<br>3 to 4<br>5 to 6<br>7 to 8<br>≥9		Repeated for each of the six reasons for laser treatment
Hair dyeing	All	Yes<br>No	x	Included dyeing, toning, and bleaching
Frequency last 5 years	Hair dyeing status= yes	≥3 times per year<br>1 to 2 times per year<br><1 time per year		
Where hair dyeing was performed	Hair dyeing status = yes	Hair salon<br>Home<br>Other	x	
Age at first hair dyeing	Hair dyeing status = yes	<18 years<br>18 to 29 years<br>≥29 years		
Pigmentation and sun habits				
Childhood eczema	All	Yes<br>No<br>Don't know		
Atopic childhood eczema	All	Yes<br>No<br>Don't know		Atopy defined as dry and itchy skin, often with rash, bumps, and sometimes with oozing blisters

Nielsen C, *et al. BMJ Open* 2023; 13:e069664. doi: 10.1136/bmjopen-2022-069664

Cohort Profile: The Swedish Tattoo and Body Modifications Cohort (TABOO)

Natural eye colour	All	Blue<br>Gray<br>Green<br>Yellowish<br>Light brown<br>Dark brown<br>Mottled/mixed<br>Heterochromia	
Natural skin tone[1]	All	Very fair<br>Fair<br>Medium<br>Olive, light brown<br>Brown<br>Dark brown	Question with illustrated examples
Freckles after sun exposure	All	Yes, many<br>Yes, few<br>No	
Skin reaction to first sun exposure	All	Never burns but tans darker<br>Burns slightly then tans<br>Burns with pain lasting ≤1 day<br>Burns with pain lasting ≥2 days (may blister)	
Severe sunburn, <13 years	All	Yes, multiple occasions<br>Yes, single occasion<br>No	
Severe sunburn, >13 years	All	Yes, multiple occasions<br>Yes, single occasion<br>No	
Recreational sun travel, <13 years	All	Yes, yearly<br>Yes, but not regularly<br>No	
Recreational sun travel, >13 years	All	Yes, yearly<br>Yes, but not regularly<br>No	
Sunscreen use in childhood	All	Yes, always<br>Yes, sometimes<br>No<br>Don't know	
Sunscreen use in adulthood	All	Yes, always<br>Yes, sometimes<br>No<br>Don't know	
Sunbed use, <13 years	All	Yes, multiple occasions	

Nielsen C, *et al. BMJ Open* 2023; 13:e069664. doi: 10.1136/bmjopen-2022-069664

Cohort Profile: The Swedish Tattoo and Body Modifications Cohort (TABOO)

		Yes, single occasion<br>No	
Sunbed use, >13 years	All	Yes, multiple occasions<br>Yes, single occasion<br>No	
Occupational sun exposure (April-September)	All	Yes<br>No<br>Not working	
Duration of occupational sun exposure (h per week)	Occupational sun exposure = yes	≤5<br>6 to 20<br>>20	
Changed sun habits after being tattooed	Tattoo status = yes	Yes, sunbath more<br>Yes, sunbath less<br>Yes, same extent of sunbathing but cover or use sunscreen on tattooed skin<br>No	
Tobacco smoking	All	Yes, daily<br>Yes, but not daily<br>No, but previously<br>No, never	
Amount smoked per day	Tobacco smoking = yes	Cigarettes<br>E-cigarettes<br>Cigars<br>Cigarillos<br>Pipes	Numbers of each type
Age when started smoking	Tobacco smoking = yes	Years	
If quit smoking, age at quitting	Tobacco smoking = yes	Years	
Snuff use	All	Yes, daily<br>Yes, but not daily<br>No, but previously<br>No, never	Only nicotine-containing snuff
Portions per day	Snuff use = yes	Number	
Age when started snuff use	Snuff use = yes	Years	
If quit snuff use, age at quitting	Snuff use = yes	Years	
Alcohol consumption frequency	All	Daily<br>4-6 days per week<br>2-3 days per week<br>1 day per week<br>2-3 days per month<br>Rare occasions<br>Never	Beverages with ≥3.5 % alcohol

[1]According to the Fitzpatrick Skin Phototype Classification[17].

Cohort Profile: The Swedish Tattoo and Body Modifications Cohort (TABOO)

Table S2. Diagnostic codes according to the International Classification of Diseases (ICD), versions 10 and 9, used to retrieve outcome data from the National Patient Register in 2021.

Version 10		Version 9	
ICD-10	Level of detail[1]	ICD-9	Level of detail
D86	Full	135	Full
E03-06	Full	240.0	Full
F32	Category	240.9	Full
F41	Category	241.0-1	Full
F43	Category	241.9	Full
F45.4	Full	242.0-4	Full
G43-44	Category	242.8-9	Full
G53.2	Full	243	Full
H22.1	Full	244.3	Full
I41.8	Full	244.8-9	Full
M05.9	Full	245.0-4	Full
M06.0	Full	245.8-9	Full
M06.9	Full	246.1-2	Full
M25.5	Full	246.8	Full
M63.3	Full	296.1	Category
M79.1	Full	296.9	Category
M79.6-7	Full	298.0	Category
M54	Category	300.0-4	Category
R10	Category	306.0	Full
R51	Category	307.8	Full
R52.1-2	Full	308.0-4	Category
R52.9	Full	308.9	Category
		309.0-4	Category
		309.8-9	Category
		311	Category
		346.0-2	Category
		346.8-9	Category
		352.6	Full
		364.1A	Full
		364.8	Full
		429.0	Full
		536.8	Category
		574.2	Category
		714.0	Full
		714.8	Full
		719.4	Full
		723.1	Category
		723.4	Category
		723.6	Category
		724.4	Category
		724.1-3	Category
		724.5	Category
		724.8-9	Category
		729.1-2	Full
		729.5	Full
		780.9	Full
		784.0	Category
		789.0	Category

[1]Level of detail refers to the amount of information pulled from the ICD alphanumeric code. Full is the complete set of characters, category is the first three positions.

Nielsen C, *et al. BMJ Open* 2023; 13:e069664. doi: 10.1136/bmjopen-2022-069664

Cohort Profile: The Swedish Tattoo and Body Modifications Cohort (TABOO)

Table S3. Anatomical Therapeutic Chemical (ATC) codes used to retrieve outcome data from the National Prescribed Drug Register in 2021.

ATC-code	Level of detail[1]
A05AA02	Chemical substance
D0	Chemical subgroup
D11AH	Chemical subgroup
H02-3	Chemical substance
J01	Pharmacological subgroup
J06BA	Chemical substance
L	Chemical substance
M01-2	Chemical substance
N02	Chemical subgroup
N05-6	Pharmacological subgroup
N07	Chemical subgroup
R03	Chemical subgroup
R06	Pharmacological subgroup
S01B, C, and G	Chemical subgroup
S03B, and C	Chemical subgroup

[1]Level of detail refers to the amount of information pulled from the ATC alphanumeric code.
