# Supplementary material for: Cohort profile: The Swedish Tattoo and Body Modifications Cohort (TABOO)
Source: BMJ Open. 2023 May 4;13(5):e069664. doi: 10.1136/bmjopen-2022-069664 (PMC10163470; doi:10.1136/bmjopen-2022-069664)
Supplement: Author's manuscript [file bmjopen-2022-069664.draft_revisions.pdf]

BMJ Open

BMJ Open is committed to open peer review. As part of this commitment we make the peer review history of every article we publish publicly available.

When an article is published we post the peer reviewers' comments and the authors' responses online. We also post the versions of the paper that were used during peer review. These are the versions that the peer review comments apply to.

The versions of the paper that follow are the versions that were submitted during the peer review process. They are not the versions of record or the final published versions. They should not be cited or distributed as the published version of this manuscript.

BMJ Open is an open access journal and the full, final, typeset and author-corrected version of record of the manuscript is available on our site with no access controls, subscription charges or pay-per-view fees (http://bmjopen.bmj.com).

If you have any questions on BMJ Open's open peer review process please email info.bmjopen@bmj.com

BMJ Open

Cohort Profile: The Swedish Tattoo and Body Modifications Cohort (TABOO)

Journal:	*BMJ Open*
Manuscript ID	bmjopen-2022-069664
Article Type:	Cohort profile
Date Submitted by the Author:	28-Oct-2022

Complete List of Authors:	Nielsen, Christel; Lund University, Laboratory Medicine, Occupational and Environmental Medicine; University of Southern Denmark, Clinical Pharmacology, Pharmacy and Environmental Medicine Department of Public Health<br>Andréasson, Kristofer; Lund University, Clinical Sciences, Rheumatology<br>Olsson, H; Lund University, Clinical Sciences, Cancer Epidemiology<br>Engfeldt, Malin; Lund University, Laboratory Medicine, Occupational and Environmental Medicine; Region Skåne, Occupational and Environmental Medicine<br>Jöud, Anna; Lund University, Laboratory Medicine, Occupational and Environmental Medicine; Region Skåne, Skåne University Hospital, Research and Education

Keywords:	EPIDEMIOLOGY, PUBLIC HEALTH, STATISTICS & RESEARCH METHODS

SCHOLARONE™
Manuscripts

I, the Submitting Author has the right to grant and does grant on behalf of all authors of the Work (as defined in the below author licence), an exclusive licence and/or a non-exclusive licence for contributions from authors who are: i) UK Crown employees; ii) where BMJ has agreed a CC-BY licence shall apply, and/or iii) in accordance with the terms applicable for US Federal Government officers or employees acting as part of their official duties; on a worldwide, perpetual, irrevocable, royalty-free basis to BMJ Publishing Group Ltd ("BMJ") its licensees and where the relevant Journal is co-owned by BMJ to the co-owners of the Journal, to publish the Work in this journal and any other BMJ products and to exploit all rights, as set out in our licence.

The Submitting Author accepts and understands that any supply made under these terms is made by BMJ to the Submitting Author unless you are acting as an employee on behalf of your employer or a postgraduate student of an affiliated institution which is paying any applicable article publishing charge ("APC") for Open Access articles. Where the Submitting Author wishes to make the Work available on an Open Access basis (and intends to pay the relevant APC), the terms of reuse of such Open Access shall be governed by a Creative Commons licence – details of these licences and which Creative Commons licence will apply to this Work are set out in our licence referred to above.

Other than as permitted in any relevant BMJ Author's Self Archiving Policies, I confirm this Work has not been accepted for publication elsewhere, is not being considered for publication elsewhere and does not duplicate material already published. I confirm all authors consent to publication of this Work and authorise the granting of this licence.

For peer review only

COHORT PROFILE

Cohort Profile: The Swedish Tattoo and Body Modifications Cohort (TABOO)

Authors: Christel Nielsen[1,2], Kristofer Andréasson[3], Håkan Olsson[,4], Malin Engfeldt[1,5], Anna Jöud[1,6]

[1]Division of Occupational and Environmental Medicine, Lund University, Lund, Sweden

[2]Clinical Pharmacology, Pharmacy and Environmental Medicine, Department of Public Health, University of Southern Denmark, Odense, Denmark

[3]Division of Rheumatology, Department of Clinical Sciences, Lund University, Lund, Sweden

[4]Division of Cancer Epidemiology, Department of Clinical Sciences, Lund University, Lund, Sweden

[5]Department of Occupational and Environmental Medicine, Region Skåne, Lund, Sweden

[6]Department of Research and Education, Skåne University Hospital, Lund, Sweden

Author deceased.

Corresponding author: Christel Nielsen
Lund University
Division of Occupational and Environmental Medicine,
Department of Laboratory Medicine
Medicon Village (402A), Scheelevägen 8
SE-223 81 Lund
Sweden
Email: christel.nielsen@med.lu.se; Phone: +46 46 2221798

For peer review only - http://bmjopen.bmj.com/site/about/guidelines.xhtml

COHORT PROFILE

Abstract

Purpose: The TABOO cohort was established to provide an infrastructure for epidemiologic studies researching the role of tattoos and other body modifications as risk factors for adverse health outcomes. It is the first population-based cohort with detailed exposure assessment of decorative, cosmetic, and medical tattoos, piercing, scarification, henna tattoos, cosmetic laser treatments, hair dyeing, and sun habits. The level of detail in the exposure assessment of tattoos allows for investigation of dose-response relationships.

Participants: The TABOO cohort includes 13,049 individuals that participated in a questionnaire survey conducted in 2021 (response rate 49 %). Outcome data is retrieved from the National Patient Register, the National Drug Register, and the National Cause of Death Register. Participation in the registers is regulated by Swedish law, which eliminates the risk of loss to follow-up and associated selection bias.

Findings to date: Several studies based on the TABOO cohort are ongoing. The cohort is used to clarify the incidence of both acute health complaints after tattooing and long-lasting conditions based on self-reported data. Using register-based outcome data, we are investigating the role of tattoos as a risk factor for immune-mediated disease, including hypersensitization, foreign body reactions, and autoimmune conditions.

Future plans: The register linkage will be renewed every third year to update the outcome data, and we have ethical approval to reapproach the responders with additional questionnaires.

For peer review only

For peer review only - http://bmjopen.bmj.com/site/about/guidelines.xhtml

COHORT PROFILE

Key words: Tattoo, body modifications, population-based cohort, register-based research, questionnaire survey

Word count: 2107

For peer review only

Strengths and limitations of this study

- TABOO is the first population-based cohort with detailed exposure assessment of tattoos and other body modifications. It was established to investigate the potential role of body modifications as risk factors for adverse health outcomes.
- Exposure was assessed at baseline in 2021 using a structured questionnaire administered by Statistics Sweden (*n* = 26,751; response rate 49 %). The cohort contains 13,049 participants [7330 females and 5719 males; ages 20-74 years].
- The exposure assessment covered decorative, cosmetic, and medical tattoos, piercing, scarification, henna tattoos, cosmetic laser treatments, hair dyeing, and sun habits, as well as personal characteristics including skin type, tobacco, and alcohol use.
- Outcome data is retrieved from the National Patient Register, the National Drug Register, and the National Cause of Death Register. Participation in the registers is regulated by Swedish law and there is no risk of loss to follow-up. Information on socio-demographic variables, including occupational history, is obtained from the Longitudinal integration database for health insurance and labour market studies. The first register linkage was performed in 2021, and we will update the outcome data every three years.

For peer review only - http://bmjopen.bmj.com/site/about/guidelines.xhtml

COHORT PROFILE

Introduction

Tattoos have become a mainstream phenomenon over the last decades. In the EU, it is estimated that 12 % of the general population, corresponding to 60 million individuals, is tattooed [1] and that most people get their first tattoo at a young age [2]. The high prevalence of tattoos and the life-long exposure to tattoo ink have brought safety concerns into the spotlight.

Tattoo inks are complex mixtures of chemicals that contain both organic and inorganic colour pigments and their by-products, as well as solvents and preservatives. Numerous reports of hazardous chemicals in tattoo inks have been published over the last years [3-5]. Coloured pigments in modern tattoo inks are dominated by azo or polycyclic compounds. This may be problematic, because some azo compounds are decomposed to toxic aromatic amines when exposed to solar radiation or laser treatment [6-8]. Then major constituent of black ink is Carbon Black, accompanied by polycyclic aromatic hydrocarbons (PAH) [1], compounds that are classified as carcinogenic or potentially carcinogenic by IARC [9, 10].

During the tattooing process, large amounts of pigment are injected into the dermis, causing damage to the skin and triggering the immune system. The immediate response involves a local reaction induced by immunological cells. As injected pigment is phagocytized by immune cells, it is transported to local lymph nodes where it initiates a systemic immune response. Deposition of tattoo pigment in human lymph nodes has been confirmed [11, 12], and the liver, spleen, and kidneys have been proposed as other plausible destinations [13]. Although research is limited, deposition of tattoo pigment in the liver has been established in mice [14]. Thus, pigment exposure is not isolated to the tattoo site, but is systemic and may continue over months or even years as pigments decompose [13].

For peer review only

For peer review only - http://bmjopen.bmj.com/site/about/guidelines.xhtml

Despite a high prevalence of tattoos, improved understanding of the chemical composition of pigments, and an urgent need to understand how tattoos may affect public health, epidemiological studies of adverse health outcomes are lacking. A limiting factor has been the absence of a sufficiently large cohort with adequate exposure assessment.

We performed three case-control studies, leveraging national Swedish registers and a questionnaire developed specifically for exposure assessment of tattoos, to investigate potential associations between tattoos and malignant melanoma, cutaneous squamous-cell carcinoma, and lymphoma. The survey was performed in 2021. The Swedish Tattoo and Body Modifications Cohort (TABOO) was established by pooling the *controls* from these studies. It constitutes the largest population-based cohort with detailed exposure assessment of tattoos and other body modifications as well as adverse health outcomes.

Cohort description

Setting

The TABOO cohort uses questionnaires for exposure assessment and leverages administrative Health Care Registers to obtain outcome data. Information on potential confounders is retrieved from both questionnaires and registers.

Participation in Swedish Health Care Registers is regulated by the Act on Health Data Registers (1998:543) and several regulations (i.e., 2001:707; 2001:709; 2005:363). Thus, they have full population coverage and there is no risk of selection bias. Furthermore, there is no loss to follow-up unless individuals emigrate from Sweden. Data sources are merged based on personal identity numbers, which are unique and permanent throughout life [15].

For peer review only - http://bmjopen.bmj.com/site/about/guidelines.xhtml

Participants

In the original case-control studies, 3000 primary cases aged 20 to 60 years at the date of diagnosis were identified for each cancer type by the National Board of Health and Welfare from the National Cancer Register in 2020. The index date ranged from 2007 to 2017, depending on the incidence of the specific neoplasm. Statistics Sweden identified three random, but sex-matched, controls per case from the Total Population Register using incidence-density sampling with age as the underlying time scale. Individuals with other malignancies than malignant melanoma, squamous-cell carcinoma in the skin, and lymphoma were eligible as controls. The study population was cross-referenced with the National Patient Register to exclude vulnerable individuals according to prespecified psychiatric diagnostic codes (i.e., F20-29 in the International Statistical Classification of Diseases and Related Health Problems, 10th Revision).

In total, 26,751 controls were invited to participate and the overall response rate among controls was 49 %, implying a final cohort size after pooling of 13,049 participants. Baseline characteristics of responders and non-responders are presented in Table 1. Responders were generally older, married individuals born in Sweden with higher educational attainment and income. There was a slight overweight towards female participants.

For peer review only - http://bmjopen.bmj.com/site/about/guidelines.xhtml

COHORT PROFILE

Table 1. Socio-demographic characteristics of the 13,049 responders and the 13,702 non-responders at baseline in 2021.

	Responders		Non-responders	
	n	(%)[1]	*n*	(%)
Sex				
Male	5719	45	7116	55
Female	7330	53	6586	47
Age				
20-29	167	28	437	72
30-39	788	34	1510	66
40-49	1714	40	2622	61
50-59	4685	49	4859	51
60-69	5692	57	4271	43
70-79	3	50	3	50
Educational attainment				
Primary and lower secondary	1061	32	2222	68
Upper secondary	5724	45	6916	55
Post-secondary	6243	60	4348	41
Missing	21	9	216	91
Country of birth				
Sweden	11,393	53	9951	47
Other	1656	31	3751	69
Citizenship				

For peer review only

For peer review only - http://bmjopen.bmj.com/site/about/guidelines.xhtml

Swedish	12,711	50	12,699	50
Dual	338	25	1003	75
Marital status				
Married	7317	55	6051	45
Unmarried	3576	43	4828	57
Divorced	1941	43	2586	57
Widowed	215	48	237	52
Disposable income (SEK)				
None (0)	248	25	743	75
1 – 124,999	586	30	1342	70
125,000 – 199,999	802	39	1243	61
200,000 – 279,999	1385	44	1793	56
280,000 – 369,999	2866	47	3221	53
≥370,000	7162	57	5360	43

[1]Percentages not summing to 100 are caused by rounding.

Exposure assessment

Self-reported exposure status was assessed through a questionnaire survey, administered by Statistics Sweden, between February and April 2021. The questionnaire was titled *'The role of new life-style factors in skin cancer, lymphoma, and other diseases'* to avoid bias from self-selection related to exposure status.

The questionnaire could be answered either on the web or on paper. The initial contact with the study population was through regular mail, where they received information about the study and log-in instructions to the digital questionnaire. Participants were informed that they

For peer review only - http://bmjopen.bmj.com/site/about/guidelines.xhtml

consented to participation if they answered the questionnaire. Three postal reminders were made. The timeline of the data-collection process is described in Table 2.

Table 2. Data-collection process and in-flow of responses among controls in the questionnaire survey.

Dispatch	Content	Date	Responders (*n*)	Response rate (%)
1	Study information<br>Log-in instruction	2 February	6530	24.4
2	Log-in instruction	16 February	1907	7.1
3	Log-in instruction<br>Paper questionnaire	2 March	3488	13.0
4	Log-in instruction	16 March	1124	4.2
Total		2 February - 16 April	13,049	48.8

The questionnaire comprised 143 questions. Statistics Sweden performed a metrological assessment of the questionnaire's disposition, question formulation, and response options, to identify potential problems, analyse their consequences, and provide solutions. After revision, the questionnaire was piloted on 28 tattooed individuals, including a tattoo artist, for content and clarity.

The questionnaire asked about tattoo status and characteristics, scarification, piercing, hair dyeing, henna tattoos, and cosmetic laser treatments (including, but not limited to, tattoo removal). It addressed sun habits according to validated questions previously used in the

For peer review only - http://bmjopen.bmj.com/site/about/guidelines.xhtml

For peer review only

COHORT PROFILE

Melanoma Inquiry of Southern Sweden (MISS) cohort [16], covering recreational and occupational sun habits, history of sunburns, use of tanning beds, and use of sunscreen. We also asked whether sun habits had changed after getting tattooed. Finally, we asked about skin characteristics, i.e., pigmentation and reaction to sun exposure, and life-style factors such as smoking, snuff use, and alcohol consumption. A full description of the exposure variables is given in Table 3.

The questionnaire defined tattoos as *permanent* body art obtained for decorative, cosmetic (i.e., permanent, or semi-permanent makeup, and microblading), or medical (i.e., skin reconstruction after surgery) purpose. Respondents were explicitly asked to consider also removed tattoos. Exposure was assessed as a dichotomous variable, i.e., presence or absence of at least one tattoo. To allow investigations of dose-response relationships, we assessed the area of tattooed body surface as:

- Increments on ordinal scale (i.e., less than 1 palm, 1 to 5 palms, or more than 5 palms),
- Number of tattoos (≥20 cm to another tattoo),
- Number of tattoo sessions, and
- Anatomical site of tattoos (according to a body manikin; Figure 1).

For peer review only

COHORT PROFILE

Figure 1. Body manikin used to retrieve information on anatomical site and approximate size of tattooed body surface.

The reliability of the exposure assessment was assessed in the pilot study. The pilot participants and a trained research assistant assessed the area of tattooed body surface at the same occasion. The weighted Cohen's kappa for the ordinal variable was estimated at 0.79 (95 % confidence interval: 0.62-0.95), implying strong inter-rater agreement.

For peer review only

For peer review only - http://bmjopen.bmj.com/site/about/guidelines.xhtml

COHORT PROFILE

Table 3. Exposure data and personal characteristics collected through the TABOO questionnaire.

Variable	Who got the question	Response options	Multiple responses possible	Comment
Tattoos				
Tattoo status	All	Yes (including any removed tattoo)<br>No		
Type of tattoo	Tattoo status = yes	Decorative<br>Cosmetic<br>Medical	x	
Age at first tattoo	Tattoo status = yes	Years		
Age at last tattoo	Tattoo status = yes	Years		
Tattooed anatomical sites	Tattoo status = yes	1: Head<br>2: Neck<br>3: Right upper arm, chest, upper back<br>4: Left upper arm, chest, upper back<br>5: Right lower arm, hand	x	The numbered response options refer to anatomical sites in Figure 1, which was included in

For peer review only - http://bmjopen.bmj.com/site/about/guidelines.xhtml

		6: Left lower arm, hand		the questionnaire
		7: Abdomen, groin, genitals, lower back, buttocks		
		8: Right thigh, knee		
		9: Left thigh, knee		
		10: Right lower leg, foot		
		11: Left lower leg, foot		
Area of tattooed body surface	Tattoo status = yes	<1 hand		
		1 to 5 hands		
		>5 hands		
By whom and where tattoo was made	Tattoo status = yes	Professional tattoo artist in tattoo studio	x	
		Professional tattoo artist at other facility		
		Cosmetic tattoo artist in cosmetic		

For peer review only

For peer review only - http://bmjopen.bmj.com/site/about/guidelines.xhtml

		tattoo studio, beauty salon, or plastic surgery clinic<br>Health care professional at hospital or other health care establishment<br>Other irrespective of where	
Number of tattoos	Tattoo status = yes	1<br>2 to 3<br>4 to 5<br>6 to 9<br>≥10	Tattoos considered separate if ≥20 cm between them<br>Cover-ups counted as 2 tattoos
Number of tattoo sessions	Tattoo status = yes	1<br>2 to 3<br>4 to 5<br>6 to 9	

For peer review only

For peer review only - http://bmjopen.bmj.com/site/about/guidelines.xhtml

COHORT PROFILE

		≥10	
Tattooed under the influence of alcohol or drugs	Tattoo status = yes	Yes	
		No	
Tattoo colours	Tattoo status = yes	Black	x
		Gray	
		Brown	
		red	
		Blue	
		Green	
		Yellow	
		White	
		Purple	
		Pink	
		Orange	
		Turquoise	
		Skin tone (injected colour)	
		Other	
Geographical regions of tattooing	Tattoo status = yes	Sweden	x
		Nordic countries (not Sweden)	
		Rest of Europe (not Nordic countries)	

For peer review only

		Asia<br>Oceania<br>USA<br>Other		
Other body modifications				
Piercing status	All	Yes<br>No, had earlier<br>No, never		Including earlobes
Scarification status	All	Yes<br>No		Scars obtained from branding or cutting
Henna tattoo status	All	Yes, black<br>Yes, red<br>No	x	Tattoo painted on skin
Laser treatment: Tattoo removal	Tattoo status = yes	Yes<br>No		
Laser treatment: Hair removal	All	Yes<br>No		Intense pulsed light (IPL given as an example)
Laser treatment: Mole or	All	Yes<br>No		Intense pulsed light

For peer review only

hyperpigmentation removal				(IPL given as an example)
Laser treatment: Thread vein removal	All	Yes<br>No		Intense pulsed light (IPL given as an example)
Laser treatment: Facial wrinkles reduction	All	Yes<br>No		Intense pulsed light (IPL given as an example)
Laser treatment: Other reason	All	Yes<br>No		Intense pulsed light (IPL given as an example)
Anatomical site of treatment	Laser treatment (specified) = yes	Face<br>Arms<br>Legs<br>Back<br>Chest and/or abdomen<br>Other	x	Repeated for each of the six reasons for laser treatment
Where laser treatment was performed	Laser treatment (specified) = yes	Beauty salon, beauty clinic, or plastic surgery clinic		Repeated for each of the six reasons

For peer review only

For peer review only - http://bmjopen.bmj.com/site/about/guidelines.xhtml

		Health care establishment<br>Home<br>Other<br>(For tattoo removal, the option tattoo artist was provided)		for laser treatment
Number of laser treatments	Laser treatment (specified) = yes	1 to 2<br>3 to 4<br>5 to 6<br>7 to 8<br>≥9		Repeated for each of the six reasons for laser treatment
Hair dyeing status	All	Yes<br>No	x	Included dyeing, toning, and bleaching
Frequency last 5 years	Hair dyeing status= yes	≥3 times per year<br>1 to 2 times per year<br><1 time per year		
Where hair dyeing was performed	Hair dyeing status = yes	Hair salon<br>Home	x	

For peer review only

COHORT PROFILE

		Other	
Age at first hair dyeing	Hair dyeing status = yes	<18 years	
		18 to 29 years	
		≥29 years	
Personal characteristics			
Childhood eczema	All	Yes	
		No	
		Don't know	
Atopic childhood eczema	All	Yes	Atopy defined as dry and itchy skin, often with rash, bumps, and sometimes with oozing blisters
		No	
		Don't know	
Natural eye colour	All	Blue	
		Gray	
		Green	
		Yellowish	
		Light brown	
		Dark brown	
		Mottled/mixed	

For peer review only - http://bmjopen.bmj.com/site/about/guidelines.xhtml

COHORT PROFILE

		Heterochromia	
Natural skin tone[1]	All	Very fair<br>Fair<br>Medium<br>Olive, light brown<br>Brown<br>Dark brown	Question with illustrated examples
Freckles after sun exposure	All	Yes, many<br>Yes, few<br>No	
Skin reaction to first sun exposure	All	Never burns but tans darker<br>Burns slightly then tans<br>Burns with pain lasting ≤1 day<br>Burns with pain lasting ≥2 days (may blister)	
Severe sunburn, <13 years	All	Yes, multiple occasions<br>Yes, single occasion	

For peer review only - http://bmjopen.bmj.com/site/about/guidelines.xhtml

COHORT PROFILE

		No
Severe sunburn, >13 years	All	Yes, multiple occasions<br>Yes, single occasion<br>No
Recreational sun travel, <13 years	All	Yes, yearly<br>Yes, but not regularly<br>No
Recreational sun travel, >13 years	All	Yes, yearly<br>Yes, but not regularly<br>No
Sunscreen use in childhood	All	Yes, always<br>Yes, sometimes<br>No<br>Don't know
Sunscreen use in adulthood	All	Yes, always<br>Yes, sometimes<br>No<br>Don't know
Sunbed use, <13 years	All	Yes, multiple occasions

For peer review only - http://bmjopen.bmj.com/site/about/guidelines.xhtml

COHORT PROFILE

		Yes, single occasion
		No
Sunbed use, >13 years	All	Yes, multiple occasions
		Yes, single occasion
		No
Occupational sun exposure (April-September)	All	Yes
		No
		Not working
Duration of occupational sun exposure (h per week)	Occupational sun exposure = yes	≤5
		6 to 20
		>20
Changed sun habits after being tattooed	Tattoo status = yes	Yes, sunbath more
		Yes, sunbath less
		Yes, same extent of sunbathing but cover or use sunscreen on tattooed skin
		No

For peer review only - http://bmjopen.bmj.com/site/about/guidelines.xhtml

Tobacco smoking	All	Yes, daily<br>Yes, but not daily<br>No, but previously<br>No, never	
Amount smoked per day	Tobacco smoking = yes	Cigarettes<br>E-cigarettes<br>Cigars<br>Cigarillos<br>Pipes	Numbers of each type
Age when started smoking	Tobacco smoking = yes	Years	
If quit smoking, age at quitting	Tobacco smoking = yes	Years	
Snuff use	All	Yes, daily<br>Yes, but not daily<br>No, but previously<br>No, never	Only nicotine-containing snuff
Portions per day	Snuff use = yes	Number	
Age when started snuff use	Snuff use = yes	Years	

For peer review only - http://bmjopen.bmj.com/site/about/guidelines.xhtml
For peer review only

COHORT PROFILE

If quit snuff use, age at quitting	Snuff use = yes	Years	
Alcohol consumption frequency	All	Daily<br>4-6 days per week<br>2-3 days per week<br>1 day per week<br>2-3 days per month<br>Rare occasions<br>Never	Beverages with ≥3.5 % alcohol

[1]According to the Fitzpatrick Skin Phototype Classification[17]

Outcome data

The questionnaire addressed self-reported health complaints, i.e., itch, redness, swelling, wounds and crusts, fever, papules, nodules, scaling, calluses, tenderness, pain, numbness, keloid scars, and increased sensitivity to sunlight. We distinguished between health complaints that occurred during the healing process (i.e., within one month after tattooing) and health complaints that developed with longer latency (i.e., later than one month after tattooing). We asked whether the respondent sought care for each kind of complaint, and whether he or she turned to the tattoo artist who performed the tattoo or to the health care

For peer review only - http://bmjopen.bmj.com/site/about/guidelines.xhtml

system. Similar, but less detailed, questions were asked with respect to piercing, scarification, cause-specific laser treatments. Finally, we asked whether respondents searched for information on adverse health effects before getting tattooed, and whether tattoo artists had informed about any type of health risk (related to the healing process or long-term) in connection with a tattoo session.

The health of the cohort is followed longitudinally in administrative population registers, e.g., the National Patient Register, the National Drug Register, and the National Cause of Death Register, all kept by the National Board of Health and Welfare. The registers have had national coverage since 1987, 2005, and 1961, respectively, and thus allow for both historical and prospective outcome assessment.

The first register linkage was performed in 2021. A specification of the retrieved outcome data is presented in Table 4. The specific codes according to the International Classification of Diseases (ICD) and the Anatomical Therapeutic Chemical (ATC) underlying the data selection is given in Tables S1 and S2. Outcome data will be updated regularly through renewed register linkages every three years, and there is the possibility to expand the data sources and add additional registers, variables, and new questionnaires.

TABOO has been linked to further population registers to retrieve information on potential confounders. We have obtained yearly data on highest educational attainment, primary source of income, disposable income, and marital status from the Longitudinal integration database for health insurance and labour market studies (LISA; Statistics Sweden).

For peer review only

COHORT PROFILE

Table 4. Characteristics of, and outcome data from, administrative population registers linked to the Swedish Tattoo Cohort (TABOO) in 2021.

	Content	Start year of register	Update frequency	Coverage in 2020[1]	Data linked to TABOO	Outcomes	Study period
National Patient Register	In-patient and specialized out-patient consultations to all health care professionals	In-patient: 1987<br>Out-patient: 2001	<2021: yearly<br>>2021: monthly	Specific to each diagnostic code. Examples:<br><br>Stroke 96 %[18]<br>Myocardial infarction 94 %[19]	Events with specified diagnostic codes	Sarcoidosis<br>Uveitis<br>Secondary myocarditis<br>Thyroiditis<br>Rheumatoid arthritis<br>Myositis<br>Depression<br>Anxiety<br>Stress<br>Abdominal pain<br>Headache<br>Back/neck pain<br>Joint pain/myalgia<br>Unspecified pain<br>Persistent pain	In-patient: 1990-2017<br>Out-patient: 1997-2017
					Events within specific clinics	Dermatology and venereology<br>Occupational dermatology	
National Prescribed Drug Register	Prescribed and dispensed drugs	2005	Monthly	Very high[20]	Events with specified drugs	Antibiotics<br>Corticosteroids<br>Dermatological agents	2005-2017

For peer review only - http://bmjopen.bmj.com/site/about/guidelines.xhtml

						Thyroid therapy	
						Immunotherapy	
						Immunoglobulin therapy	
						Bile therapy	
						Antiinflammatory and antirheumatic therapy	
						Topical treatments for joint pain and myalgia	
						Analgetics	
						Neuroleptics	
						Psychoanaleptics	
						Adrenergics	
						Antihistamine	
National Cause of Death Register	Deaths in Sweden and among Swedish citizens that died abroad	1961	Yearly	Very high[21]	Death	Date	2007-2017

[1]Compared with National Quality Registers.

For peer review only

For peer review only - http://bmjopen.bmj.com/site/about/guidelines.xhtml

Findings to date

Several studies based on the TABOO cohort are ongoing. The cohort is used to clarify the incidence of both acute health complaints after tattooing and long-lasting conditions based on self-reported data. Primary outcomes are infection, inflammation, and persistent pain. Using register-based outcome data, we are investigating the role of tattoos as a risk factor for immune-mediated disease, including hypersensitization (i.e., allergic contact dermatitis), foreign body reactions (i.e., sarcoidosis), and autoimmune conditions (i.e., rheumatoid arthritis and thyroid disease).

An updated list of publications based on TABOO will be published on the research group's web page: https://portal.research.lu.se/sv/organisations/epidemiology/publications/.

Strengths and limitations

The main strength of TABOO is that it is the first population-based cohort with exposure data with respect to tattoos and other body modifications and sufficient size, and hence statistical power, to investigate the role of these exposures as risk factors for different health outcomes, also those that are less common. The level of detail in the exposure assessment allows for investigation of dose-response relationships. Finally, the compulsory nature of the registers from which outcome data is retrieved eliminates the risk of loss to follow-up and the potential selection bias that would follow.

As with any register-based study, the lack of biological samples is a limitation. However, observational studies are well-suited to identify associations, generate more detailed hypotheses, and pinpoint research questions that warrants further studies.

For peer review only

Collaboration

We welcome research collaboration using the TABOO cohort. To enquire about the data, and to discuss potential collaboration, please email the lead investigator Christel Nielsen [christel.nielsen@med.lu.se].

Author statement

CN and AJ designed the study. CN, AJ, HO, KA, and ME constructed the questionnaire. CN directed the study's implementation. CN, AJ, and KA defined the ICD- and ATC-codes that were retrieved from registers, and CN and AJ selected the socio-demographic variables. CN conducted the literature review and drafted the manuscript. All authors read and approved the final manuscript. CN is guarantor for the paper.

Funding

This work was supported by the Swedish Research Council for Health, Working Life, and Welfare [2018-00864 to C.N]; the Crafoord Foundation [20180659 to C.N] and the Magnus Bergvall Foundation [201802669 to C.N].

Conflicts of interests

None declared.

For peer review only - http://bmjopen.bmj.com/site/about/guidelines.xhtml

Ethics approval

The study was approved by the Swedish Ethical Review Authority (no. 2019-03138). Ethical approval to reapproach the responders with additional questionnaires has been obtained.

Patient and public involvement

The study population was the sex- and age-matched controls to the cases in the original studies, but otherwise constituted a representative sample of the Swedish general population in terms of sociodemographic characteristics. Members of the public participated in the pilot of the survey questions, and we implemented their feedback to improve the content of and the formulations in the questionnaire.

Data availability statement

The data underlying this article cannot be shared publicly due to the privacy of the individuals that participated in the study. Register data are obtained from third parties and are not publicly available. However, data from the TABOO cohort can be made available for research upon reasonable request, and after approval from the Swedish Ethical Review Authority.

Acknowledgements

The authors are grateful to the pilot participants that provided constructive input to improve the clarity and validity of the questionnaire. We also express a big thank you to all the study

For peer review only

participants that dedicated their time and effort to help us establish a unique research infrastructure that will move the field of health effects related to body modifications forward.

References

1. Piccinini P, Pakalin S, Contor L, Bianchi I, Senaldi C. Safety of tattoos and permanent make-up: Final report. EUR 27947. Luxembourg (Luxembourg): *Publications Office of the European Union*; 2016. JRC101601.

2. Klügl I, Hiller KA, Landthaler M, Bäumler W. Incidence of health problems associated with tattooed skin: a nation-wide survey in German-speaking countries. *Dermatology* 2010;**221**:43-50.

3. Manso M, Pessanha S, Guerra M, Reinholz U, Afonso C, Radtke M, Lourenço H, Carvalho ML, Buzanich AG. Assessment of toxic metals and hazardous substances in tattoo inks using Sy-XRF, AAS, and Raman spectroscopy. *Biol Trace Elem Res* 2019;**187**:596-601.

4. Wang X, Josefsson L, Meschnark S, Lind ML, Emmer Å, Goessler W, Hedberg YS. Analytical survey of tattoo inks - a chemical and legal perspective with focus on sensitizing substances. *Contact Dermat* 2021;**85**:340-53.

5. Swedish Medical Products Agency. Kontroll av tatueringsfärger för tatuering och permanent makeup med analysprotokoll - 2020. *Rapport från Läkemedelsverket* 2020. URL: https://www.lakemedelsverket.se/491725/globalassets/dokument/publikationer/tatueringsfarger/kontroll-av-tatueringsfarger-med-analysprotokoll-2020.pdf. Accessed 2022-10-26.

For peer review only - http://bmjopen.bmj.com/site/about/guidelines.xhtml

6. Vasold R, Naarmann N, Ulrich H, Fischer D, König B, Landthaler M, Bäumler W. Tattoo pigments are cleaved by laser light-the chemical analysis *in vitro* provide evidence for hazardous compounds. *Photochem Photobiol* 2004;**80**:185-90.

7. Engel E, Spannberger A, Vasold R, König B, Landthaler M, Bäumler W. Photochemical cleavage of a tattoo pigment by UVB radiation or natural sunlight. *J Dtsch Dermatol Ges* 2007;**5**:583-9.

8. Cui Y, Spann AP, Couch LH, Gopee NV, Evans FE, Churchwell MI, Williams LD, Doerge DR, Howard PC. Photodecomposition of Pigment Yellow 74, a pigment used in tattoo inks. *Photochem Photobiol* 2004;**80**:175-84.

9. IARC working group on the evaluation of carcinogenic risks to humans. Some non-heterocyclic polycyclic aromatic hydrocarbons and some related exposures. *IARC Monogr Eval Carcinog Risks Hum* 2010;**92**:1-853.

10. IARC working group on the evaluation of carcinogenic risks to humans. Carbon black, titanium dioxide, and talc. *IARC Monogr Eval Carcinog Risks Hum* 2010;**93**:1-413.

11. Lehner K, Santarelli F, Vasold R, Penning R, Sidoroff A, König B, Landthaler M, Bäumler W. Black tattoos entail substantial uptake of genotoxicpolycyclic aromatic hydrocarbons (PAH) in human skin and regional lymph nodes. *PloS one* 2014;**9**:e92787.

12. Schreiver I, Hesse B, Seim C, Castillo-Michel H, Villanova J, Laux P, Dreiack N, Penning R, Tucoulou R, Cotte M, Luch A. Synchrotron-based *ν*-XRF mapping and *µ*-FTIR microscopy enable to look into the fate and effects of tattoo pigments in human skin. *Sci Rep* 2017;**7**:11395.

13. Bäumler W. Absorption, distribution, metabolism and excretion of tattoo colorants and ingredients in mouse and man: the known and the unknown. *Curr Probl Dermatol* 2015;**48**:176-84.

For peer review only - http://bmjopen.bmj.com/site/about/guidelines.xhtml

14. Sepehri M, Sejersen T, Qvortrup K, Lerche CM, Serup J. Tattoo pigments are observed in the Kupffer cells of the liver indicating blood-borne distribution of tattoo ink. *Dermatology* 2017;**233**:86-93.

15. Ludvigsson JF, Otterblad-Olausson P, Pettersson BU, Ekbom A. The Swedish personal identity number: possibilities and pitfalls in healthcare and medical research. *Eur J Epidemiol* 2009;**24**:659-67.

16. Westerdahl J, Anderson H, Olsson H, Ingvar C. Reproducibility of a self-administered questionnaire for assessment of melanoma risk. *Int J Epidemiol* 1996;**25**:245-51.

17. Fitzpatrick TB. The validity and practicality of sun-reactive skin types I through VI. *Arch Dermatol* 1988;**124**:869-71.

18. Engdahl B. Kvalitetsdeklaration: Statistik om hjärtinfarkter 2020. *Socialstyrelsen* 2021. URL: https://www.socialstyrelsen.se/globalassets/sharepoint-dokument/artikelkatalog/statistik/2021-12-7648-kvalitetsdeklaration.pdf. Accessed 2022-10-26.

19. Lovering C. Kvalitetsdeklaration: statistik om stroke 2020. *Socialstyrelsen* 2021. URL: https://www.socialstyrelsen.se/globalassets/sharepoint-dokument/artikelkatalog/statistik/2021-12-7643-kvalitetsdeklaration.pdf. Accessed 2022-10-26.

20. Ohlin M, Otterdal P. Det statistiska registrets framställning och kvalitet: läkemedelsregistret. *Socialstyrelsen* 2021. URL: https://www.socialstyrelsen.se/globalassets/sharepoint-dokument/dokument-webb/statistik/statistiska-registrets-framstallning-kvalitet-lakemedelsregistret.pdf. Accessed 2022-10-26.

For peer review only

For peer review only - http://bmjopen.bmj.com/site/about/guidelines.xhtml

COHORT PROFILE

21. de Munter J. Det statistiska registrets framställning och kvalitet: dödsorsaksregistret. *Socialstyrelsen* 2022. URL: https://www.socialstyrelsen.se/globalassets/sharepoint-dokument/artikelkatalog/statistik/2022-5-7909.pdf. Accessed 2022-10-26.

For peer review only

For peer review only - http://bmjopen.bmj.com/site/about/guidelines.xhtml

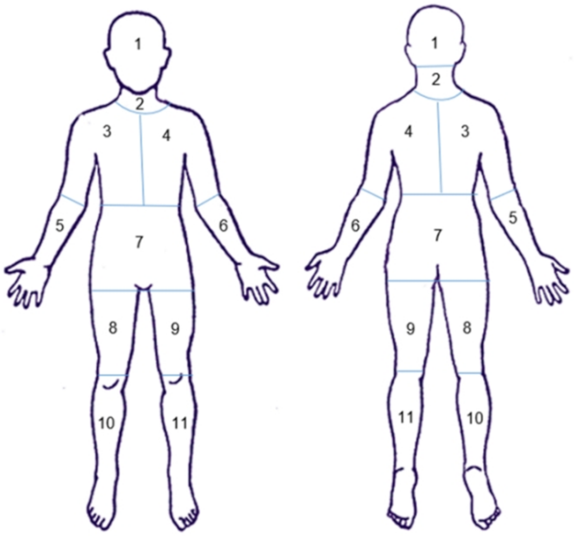

Figure 1. Body manikin used to retrieve information on anatomical site and approximate size of tattooed body surface.

855x481mm (38 x 38 DPI)

COHORT PROFILE: THE SWEDISH TATTOO AND BODY MODIFICATIONS COHORT (TABOO)

Supplementary material

Table S1. Diagnostic codes according to the International Classification of Diseases (ICD), versions 10 and 9, used to retrieve outcome data from the National Patient Register in 2021.

Version 10		Version 9	
ICD-10	Level of detail[1]	ICD-9	Level of detail
D86	Full	135	Full
E03-06	Full	240.0	Full
F32	Category	240.9	Full
F41	Category	241.0-1	Full
F43	Category	241.9	Full
F45.4	Full	242.0-4	Full
G43-44	Category	242.8-9	Full
G53.2	Full	243	Full
H22.1	Full	244.3	Full
I41.8	Full	244.8-9	Full
M05.9	Full	245.0-4	Full
M06.0	Full	245.8-9	Full
M06.9	Full	246.1-2	Full
M25.5	Full	246.8	Full
M63.3	Full	296.1	Category
M79.1	Full	296.9	Category
M79.6-7	Full	298.0	Category
M54	Category	300.0-4	Category
R10	Category	306.0	Full
R51	Category	307.8	Full
R52.1-2	Full	308.0-4	Category
R52.9	Full	308.9	Category
		309.0-4	Category
		309.8-9	Category
		311	Category
		346.0-2	Category
		346.8-9	Category
		352.6	Full
		364.1A	Full
		364.8	Full
		429.0	Full
		536.8	Category
		574.2	Category
		714.0	Full
		714.8	Full
		719.4	Full
		723.1	Category
		723.4	Category
		723.6	Category
		724.4	Category
		724.1-3	Category
		724.5	Category
		724.8-9	Category
		729.1-2	Full
		729.5	Full
		780.9	Full
		784.0	Category
		789.0	Category

[1]Level of detail refers to the amount of information pulled from the ICD alphanumeric code. Full is the complete set of characters, category is the first three positions.

For peer review only

COHORT PROFILE: THE SWEDISH TATTOO AND BODY MODIFICATIONS COHORT (TABOO)

Table S2. Anatomical Therapeutic Chemical (ATC) codes used to retrieve outcome data from the National Prescribed Drug Register in 2021.

ATC-code	Level of detail[1]
A05AA02	Chemical substance
D0	Chemical subgroup
D11AH	Chemical subgroup
H02-3	Chemical substance
J01	Pharmacological subgroup
J06BA	Chemical substance
L	Chemical substance
M01-2	Chemical substance
N02	Chemical subgroup
N05-6	Pharmacological subgroup
N07	Chemical subgroup
R03	Chemical subgroup
R06	Pharmacological subgroup
S01B, C, and G	Chemical subgroup
S03B, and C	Chemical subgroup

[1]Level of detail refers to the amount of information pulled from the ATC alphanumeric code.

For peer review only

BMJ Open

Cohort Profile: The Swedish Tattoo and Body Modifications Cohort (TABOO)

Journal:	*BMJ Open*
Manuscript ID	bmjopen-2022-069664.R1
Article Type:	Cohort profile
Date Submitted by the Author:	14-Mar-2023
Complete List of Authors:	Nielsen, Christel; Lund University, Laboratory Medicine, Occupational and Environmental Medicine; University of Southern Denmark, Clinical Pharmacology, Pharmacy and Environmental Medicine Department of Public Health<br>Andréasson, Kristofer; Lund University, Clinical Sciences, Rheumatology<br>Olsson, H; Lund University, Clinical Sciences, Cancer Epidemiology<br>Engfeldt, Malin; Lund University, Laboratory Medicine, Occupational and Environmental Medicine; Region Skåne, Occupational and Environmental Medicine<br>Jöud, Anna; Lund University, Laboratory Medicine, Occupational and Environmental Medicine; Region Skåne, Skåne University Hospital, Research and Education
**Primary Subject Heading**:	Epidemiology
Secondary Subject Heading:	Public health
Keywords:	EPIDEMIOLOGY, PUBLIC HEALTH, STATISTICS & RESEARCH METHODS

SCHOLARONE™ Manuscripts

I, the Submitting Author has the right to grant and does grant on behalf of all authors of the Work (as defined in the below author licence), an exclusive licence and/or a non-exclusive licence for contributions from authors who are: i) UK Crown employees; ii) where BMJ has agreed a CC-BY licence shall apply, and/or iii) in accordance with the terms applicable for US Federal Government officers or employees acting as part of their official duties; on a worldwide, perpetual, irrevocable, royalty-free basis to BMJ Publishing Group Ltd ("BMJ") its licensees and where the relevant Journal is co-owned by BMJ to the co-owners of the Journal, to publish the Work in this journal and any other BMJ products and to exploit all rights, as set out in our licence.

The Submitting Author accepts and understands that any supply made under these terms is made by BMJ to the Submitting Author unless you are acting as an employee on behalf of your employer or a postgraduate student of an affiliated institution which is paying any applicable article publishing charge ("APC") for Open Access articles. Where the Submitting Author wishes to make the Work available on an Open Access basis (and intends to pay the relevant APC), the terms of reuse of such Open Access shall be governed by a Creative Commons licence – details of these licences and which Creative Commons licence will apply to this Work are set out in our licence referred to above.

Other than as permitted in any relevant BMJ Author's Self Archiving Policies, I confirm this Work has not been accepted for publication elsewhere, is not being considered for publication elsewhere and does not duplicate material already published. I confirm all authors consent to publication of this Work and authorise the granting of this licence.

For peer review only

COHORT PROFILE

Cohort Profile: The Swedish Tattoo and Body Modifications Cohort (TABOO)

Authors: Christel Nielsen[1,2], Kristofer Andréasson[3], Håkan Olsson[[illegible],4], Malin Engfeldt[1,5], Anna Jöud[1,6]

[1]Division of Occupational and Environmental Medicine, Lund University, Lund, Sweden

[2]Clinical Pharmacology, Pharmacy and Environmental Medicine, Department of Public Health, University of Southern Denmark, Odense, Denmark

[3]Division of Rheumatology, Department of Clinical Sciences, Lund University, Lund, Sweden

[4]Division of Cancer Epidemiology, Department of Clinical Sciences, Lund University, Lund, Sweden

[5]Department of Occupational and Environmental Medicine, Region Skåne, Lund, Sweden

[6]Department of Research and Education, Skåne University Hospital, Lund, Sweden

[illegible]Author deceased.

Corresponding author: Christel Nielsen

Lund University

Division of Occupational and Environmental Medicine,

Department of Laboratory Medicine

Medicon Village (402A), Scheelevägen 8

SE-223 81 Lund

Sweden

Email: christel.nielsen@med.lu.se; Phone: +46 46 2221798

For peer review only

For peer review only - http://bmjopen.bmj.com/site/about/guidelines.xhtml

COHORT PROFILE

Abstract

Purpose: The TABOO cohort was established to provide an infrastructure for epidemiologic studies researching the role of tattoos and other body modifications as risk factors for adverse health outcomes. It is the first population-based cohort with detailed exposure assessment of decorative, cosmetic, and medical tattoos, piercing, scarification, henna tattoos, cosmetic laser treatments, hair dyeing, and sun habits. The level of detail in the exposure assessment of tattoos allows for investigation of crude dose-response relationships.

Participants: The TABOO cohort includes 13,049 individuals that participated in a questionnaire survey conducted in 2021 (response rate 49 %). Outcome data is retrieved from the National Patient Register, the National Drug Register, and the National Cause of Death Register. Participation in the registers is regulated by Swedish law, which eliminates the risk of loss to follow-up and associated selection bias.

Findings to date: The tattoo prevalence in TABOO is 21 %. The cohort is currently used to clarify the incidence of both acute health complaints after tattooing and long-lasting conditions based on self-reported data. Using register-based outcome data, we are investigating the role of tattoos as a risk factor for immune-mediated disease, including hypersensitization, foreign body reactions, and autoimmune conditions.

Future plans: The register linkage will be renewed every third year to update the outcome data, and we have ethical approval to reapproach the responders with additional questionnaires.

For peer review only - http://bmjopen.bmj.com/site/about/guidelines.xhtml

COHORT PROFILE

Key words: Tattoo, body modifications, population-based cohort, register-based research, questionnaire survey

Word count: 2107

For peer review only

For peer review only - http://bmjopen.bmj.com/site/about/guidelines.xhtml

Strengths and limitations of this study

- TABOO is the first population-based cohort specifically designed to study health effects of tattoos and other body modifications.
- Exposure was assessed at baseline in 2021 using a structured questionnaire administered by Statistics Sweden (response rate 49 %).
- The cohort contains 13,049 participants and the tattoo prevalence is 21 %.
- The exposure assessment covers permanent tattoos (decorative, cosmetic, and medical), piercing, scarification, henna tattoos, cosmetic laser treatments, hair dyeing, and sun habits.
- Outcome data is available from questionnaires and from National Authority Registers with full population coverage.

review only

For peer review only - http://bmjopen.bmj.com/site/about/guidelines.xhtml

COHORT PROFILE

Introduction

Tattoos have become a mainstream phenomenon over the last decades. In the EU, it is estimated that 12 % of the general population, corresponding to 60 million individuals, is tattooed [1] and that most people get their first tattoo at a young age [2]. The high prevalence of tattoos and the life-long exposure to tattoo ink have brought safety concerns into the spotlight.

Tattoo inks are complex mixtures of chemicals that contain both organic and inorganic colour pigments and their by-products, as well as solvents, preservatives, and contaminants. Numerous reports of hazardous chemicals in tattoo inks have been published over the last years [3-5]. Coloured pigments in modern tattoo inks are dominated by azo or polycyclic compounds. This may be problematic, because some azo compounds are decomposed to toxic aromatic amines when exposed to solar radiation or laser treatment [6-8]. Then major constituent of black ink is Carbon Black, accompanied by polycyclic aromatic hydrocarbons (PAH) [1], compounds that are classified as carcinogenic or potentially carcinogenic by IARC [9, 10].

During the tattooing process, large amounts of pigment are injected into the dermis. When the skin barrier is breached and foreign bodies intrude the body, the immune system is triggered. The immediate response involves a local reaction induced by immunological cells. As foreign bodies are phagocytized by immune cells, they are transported to local lymph nodes where a systemic immune response is initiated. Deposition of tattoo pigment in human lymph nodes has been confirmed [11, 12], and the liver, spleen, and kidneys have been proposed as other plausible destinations [13]. Although research is limited, deposition of tattoo pigment in the liver has been established in mice [14]. Thus, pigment exposure is not isolated to the tattoo site, but is systemic and may continue over months or even years as pigments decompose [13].

For peer review only - http://bmjopen.bmj.com/site/about/guidelines.xhtml

Despite a high prevalence of tattoos, improved understanding of the chemical composition of pigments, and an urgent need to understand if and how tattoos affect public health, epidemiological studies of adverse health outcomes are lacking. A limiting factor has been the absence of a sufficiently large cohort with adequate exposure assessment.

We performed three case-control studies, leveraging national Swedish registers and a questionnaire developed specifically for exposure assessment of tattoos, to investigate potential associations between tattoos and malignant melanoma, cutaneous squamous-cell carcinoma, and lymphoma. The survey was performed in 2021. The Swedish Tattoo and Body Modifications Cohort (TABOO) was established by pooling the *controls* from these studies. It constitutes the largest population-based cohort with detailed exposure assessment of tattoos and other body modifications as well as adverse health outcomes.

Cohort description

Setting

The TABOO cohort uses questionnaires for exposure assessment and leverages administrative Health Care Registers to obtain outcome data. Information on potential confounders is retrieved from both questionnaires and registers.

Participation in Swedish Health Care Registers is regulated by the Act on Health Data Registers (1998:543) and several regulations (i.e., 2001:707; 2001:709; 2005:363). Thus, they have full population coverage and there is no risk of selection bias. Furthermore, there is no loss to follow-up unless individuals emigrate from Sweden. Data sources are merged based on personal identity numbers, which are unique and permanent throughout life [15].

For peer review only - http://bmjopen.bmj.com/site/about/guidelines.xhtml

COHORT PROFILE

Participants

In the original case-control studies, 3000 primary cases aged 20 to 60 years at the date of diagnosis were identified for each cancer type by the National Board of Health and Welfare from the National Cancer Register in 2020. The index date ranged from 2007 to 2017, depending on the incidence of the specific neoplasm. Statistics Sweden identified three random, but sex-matched, controls per case from the Total Population Register using incidence-density sampling with age as the underlying time scale. Individuals with other malignancies than malignant melanoma, squamous-cell carcinoma in the skin, and lymphoma were eligible as controls. The study population was cross-referenced with the National Patient Register to exclude vulnerable individuals according to prespecified psychiatric diagnostic codes (i.e., F20-29 in the International Statistical Classification of Diseases and Related Health Problems, 10th Revision).

In total, 26,751 controls were invited to participate and the overall response rate among controls was 49 %, implying a final cohort size after pooling of 13,049 participants. Baseline characteristics of responders and non-responders are presented in Table 1. Responders were generally older, married individuals born in Sweden with higher educational attainment and income compared with non-responders. There was a slight overweight towards female participants.

For peer review only - http://bmjopen.bmj.com/site/about/guidelines.xhtml

COHORT PROFILE

Table 1. Socio-demographic characteristics of the 13,049 responders and the 13,702 non-responders at baseline in 2021.

	Responders		Non-responders	
	n	(%)[1]	*n*	(%)
Sex				
Male	5719	45	7116	55
Female	7330	53	6586	47
Age				
20-29	167	28	437	72
30-39	788	34	1510	66
40-49	1714	40	2622	61
50-59	4685	49	4859	51
60-69	5692	57	4271	43
70-79	3	50	3	50
Educational attainment				
Primary and lower secondary	1061	32	2222	68
Upper secondary	5724	45	6916	55
Post-secondary	6243	60	4348	41
Missing	21	9	216	91
Country of birth				
Sweden	11,393	53	9951	47
Other	1656	31	3751	69
Citizenship				

For peer review only - http://bmjopen.bmj.com/site/about/guidelines.xhtml

For peer review only

Swedish	12,711	50	12,699	50
Dual	338	25	1003	75
Marital status				
Married	7317	55	6051	45
Unmarried	3576	43	4828	57
Divorced	1941	43	2586	57
Widowed	215	48	237	52
Disposable income (SEK)				
None (0)	248	25	743	75
1 – 124,999	586	30	1342	70
125,000 – 199,999	802	39	1243	61
200,000 – 279,999	1385	44	1793	56
280,000 – 369,999	2866	47	3221	53
≥370,000	7162	57	5360	43

[1]Percentages not summing to 100 are caused by rounding.

Exposure assessment

Self-reported exposure status was assessed through a questionnaire survey, administered by Statistics Sweden, between February and April 2021. The questionnaire was titled *'The role of new life-style factors in skin cancer, lymphoma, and other diseases'* to avoid bias from self-selection related to exposure status.

The questionnaire could be answered either on the web or on paper. The initial contact with the study population was through regular mail, where they received information about the study and log-in instructions to the digital questionnaire. Participants were informed that they

For peer review only

For peer review only - http://bmjopen.bmj.com/site/about/guidelines.xhtml

consented to participation if they answered the questionnaire. Three postal reminders were made. The timeline of the data-collection process is described in Table 2.

Table 2. Data-collection process and in-flow of responses among controls in the questionnaire survey.

Dispatch	Content	Date	Responders (n)	Response rate (%)
1	Study information<br>Log-in instruction	2 February	6530	24.4
2	Log-in instruction	16 February	1907	7.1
3	Log-in instruction<br>Paper questionnaire	2 March	3488	13.0
4	Log-in instruction	16 March	1124	4.2
Total		2 February - 16 April	13,049	48.8

The questionnaire comprised 143 questions. Statistics Sweden performed a metrological assessment of the questionnaire's disposition, question formulation, and response options, to identify potential problems, analyse their consequences, and provide solutions. After revision, the questionnaire was piloted on 28 tattooed individuals, including a tattoo artist, for content and clarity.

The questionnaire asked about tattoo status and characteristics, scarification, piercing, hair dyeing, henna tattoos, and cosmetic laser treatments (including, but not limited to, tattoo removal). It addressed sun habits according to validated questions previously used in the

For peer review only - http://bmjopen.bmj.com/site/about/guidelines.xhtml

COHORT PROFILE

Melanoma Inquiry of Southern Sweden (MISS) cohort [16], covering recreational and occupational sun habits, history of sunburns, use of tanning beds, and use of sunscreen. We also asked whether sun habits had changed after getting tattooed. Finally, we asked about skin characteristics, i.e., pigmentation and reaction to sun exposure, and life-style factors such as smoking, snuff use, and alcohol consumption. An overview of the variables in TABOO is given in Table 3. For a full description of the variables, including the possible answers and whom the questions targeted, we refer to the supplementary file.

The questionnaire defined tattoos as *permanent* body art obtained for decorative, cosmetic (i.e., permanent, or semi-permanent makeup, and microblading), or medical (i.e., skin reconstruction after surgery) purpose. Respondents were explicitly asked to consider also removed tattoos. Exposure was assessed as a dichotomous variable, i.e., presence or absence of at least one tattoo. To allow investigation of crude dose-response relationships, we assessed the area of tattooed body surface as:

- Increments on ordinal scale (i.e., less than 1 palm, 1 to 5 palms, or more than 5 palms),
- Number of tattoos (≥20 cm to another tattoo),
- Number of tattoo sessions, and
- Anatomical site of tattoos (according to a body manikin; Figure 1).

For peer review only - http://bmjopen.bmj.com/site/about/guidelines.xhtml

Figure 1. Body manikin used to retrieve information on anatomical site and approximate size of tattooed body surface.

The reliability of the exposure assessment was assessed in the pilot study. The pilot participants and a trained research assistant assessed the area of tattooed body surface at the same occasion. The weighted Cohen's kappa for the ordinal variable was estimated at 0.79 (95 % confidence interval: 0.62-0.95), implying strong inter-rater agreement.

For peer review only

Table 3. Exposure data and personal characteristics collected through the TABOO questionnaire.

Variable
Tattoos
Tattoo status
Type of tattoo (decorative, cosmetic; medical)
Age at first tattoo
Age at last tattoo
Tattooed anatomical sites
Area of tattooed body surface
By whom and where tattoo was made
Number of tattoos
Number of tattoo sessions
Tattooed under the influence of alcohol or drugs
Tattoo colours
Geographical regions of tattooing
Other body modifications
Piercing status
Scarification status
Henna tattoo status
Laser treatment: Tattoo removal
Laser treatment: Hair removal
Laser treatment: Mole or hyperpigmentation removal
Laser treatment: Thread vein removal
Laser treatment: Facial wrinkles reduction

For peer review only - http://bmjopen.bmj.com/site/about/guidelines.xhtml

Laser treatment: Other reason

Anatomical site of treatment

Where laser treatment was performed

Number of laser treatments

Hair dyeing

Frequency last 5 years

Age at first hair dyeing

Pigmentation and sun habits

Childhood eczema

Natural eye colour

Natural skin tone[1]

Freckles after sun exposure

Skin reaction to first sun exposure

Severe sunburn: <13 years; >13 years

Recreational sun travel: <13 years; >13 years

Sunscreen use: childhood; adult

Sunbed use: <13 years; >13 years

Occupational sun exposure (April-September)

Changed sun habits after being tattooed

Tobacco smoking and snuff use

Amount per day

Age when started

If quit, age at quitting

Alcohol consumption

[1]According to the Fitzpatrick Skin Phototype Classification[17].

For peer review only - http://bmjopen.bmj.com/site/about/guidelines.xhtml

For peer review only

Outcome data

The questionnaire addressed self-reported health complaints, i.e., itch, redness, swelling, wounds and crusts, fever, papules, nodules, scaling, calluses, tenderness, pain, numbness, keloid scars, and increased sensitivity to sunlight. We distinguished between health complaints that occurred during the healing process (i.e., within one month after tattooing) and health complaints that developed with longer latency (i.e., later than one month after tattooing). We asked whether the respondent sought care for each kind of complaint, and whether he or she turned to the tattoo artist who performed the tattoo or to the health care system. Similar, but less detailed, questions were asked with respect to piercing, scarification, cause-specific laser treatments. Finally, we asked whether respondents searched for information on adverse health effects before getting tattooed, and whether tattoo artists had informed about any type of health risk (related to the healing process or long-term) in connection with a tattoo session.

The health of the cohort is followed longitudinally in administrative population registers, e.g., the National Patient Register, the National Drug Register, and the National Cause of Death Register, all kept by the National Board of Health and Welfare. The registers have had national coverage since 1987, 2005, and 1961, respectively, and thus allow for both historical and prospective outcome assessment.

The first register linkage was performed in 2021. A specification of the retrieved outcome data is presented in Table 4. The specific codes according to the International Classification of Diseases (ICD) and the Anatomical Therapeutic Chemical (ATC) underlying the data selection is given in the supplementary file. Outcome data will be updated regularly through

For peer review only - http://bmjopen.bmj.com/site/about/guidelines.xhtml

renewed register linkages every three years, and there is the possibility to expand the data sources and add additional registers, variables, and new questionnaires.

TABOO has been linked to further population registers to retrieve information on potential confounders. We have obtained yearly data on highest educational attainment, primary source of income, disposable income, and marital status from the Longitudinal integration database for health insurance and labour market studies (LISA; Statistics Sweden).

For peer review only

For peer review only - http://bmjopen.bmj.com/site/about/guidelines.xhtml

COHORT PROFILE

Table 4. Characteristics of, and outcome data from, administrative population registers linked to the Swedish Tattoo Cohort (TABOO) in 2021.

	Content	Start year of register	Update frequency	Coverage in 2020[1]	Data linked to TABOO	Outcomes	Study period
National Patient Register	In-patient and specialized out-patient consultations to all health care professionals	In-patient: 1987<br>Out-patient: 2001	<2021: yearly<br>>2021: monthly	Specific to each diagnostic code. Examples:<br><br>Stroke 96 %[18]<br>Myocardial infarction 94 %[19]	Events with specified diagnostic codes	Sarcoidosis<br>Uveitis<br>Secondary myocarditis<br>Thyroiditis<br>Rheumatoid arthritis<br>Myositis<br>Depression<br>Anxiety<br>Stress<br>Abdominal pain<br>Headache<br>Back/neck pain<br>Joint pain/myalgia<br>Unspecified pain<br>Persistent pain	In-patient:<br>1990-2017<br>Out-patient:<br>1997-2017
					Events within specific clinics	Dermatology and venereology<br>Occupational dermatology	
National Prescribed Drug Register	Prescribed and dispensed drugs	2005	Monthly	Very high[20]	Events with specified drugs	Antibiotics<br>Corticosteroids<br>Dermatological agents	2005-2017

For peer review only - http://bmjopen.bmj.com/site/about/guidelines.xhtml

COHORT PROFILE

						Thyroid therapy	
						Immunotherapy	
						Immunoglobulin therapy	
						Bile therapy	
						Antiinflammatory and antirheumatic therapy	
						Topical treatments for joint pain and myalgia	
						Analgetics	
						Neuroleptics	
						Psychoanaleptics	
						Adrenergics	
						Antihistamine	
National Cause of Death Register	Deaths in Sweden and among Swedish citizens that died abroad	1961	Yearly	Very high[21]	Death	Date	2007-2017

[1]Compared with National Quality Registers.

For peer review only

Findings to date

The prevalence of tattoos in TABOO is 21 % (n=2752). TABOO is currently used to clarify the incidence of both acute health complaints after tattooing and long-lasting conditions based on self-reported data. Using register-based outcome data, we are investigating the role of tattoos as a risk factor for immune-mediated disease, including hypersensitization (i.e., allergic contact dermatitis), foreign body reactions (i.e., sarcoidosis), and autoimmune conditions (i.e., rheumatoid arthritis and thyroid disease).

An updated list of publications based on TABOO will be published on the research group's web page: https://portal.research.lu.se/sv/organisations/epidemiology/publications/.

Strengths and limitations

The main strength of TABOO is that it is the first population-based cohort with exposure data with respect to tattoos and other body modifications and sufficient size, and hence statistical power, to investigate the role of these exposures as risk factors for different health outcomes, also those that are less common. The level of detail in the exposure assessment allows for investigation of crude dose-response relationships. Finally, the compulsory nature of the registers from which outcome data is retrieved eliminates the risk of loss to follow-up and the potential selection bias that would follow.

As with any register-based study, the lack of biological samples is a limitation. However, observational studies are well-suited to identify associations, generate more detailed hypotheses, and pinpoint research questions that warrants further studies.

For peer review only - http://bmjopen.bmj.com/site/about/guidelines.xhtml

Collaboration

We welcome research collaboration using the TABOO cohort. To enquire about the data, and to discuss potential collaboration, please email the lead investigator Christel Nielsen [christel.nielsen@med.lu.se].

Author statement

CN and AJ designed the study. CN, AJ, HO, KA, and ME constructed the questionnaire. CN directed the study's implementation. CN, AJ, and KA defined the ICD- and ATC-codes that were retrieved from registers, and CN and AJ selected the socio-demographic variables. CN conducted the literature review and drafted the manuscript. All authors read and approved the final manuscript. CN is guarantor for the paper.

Funding

This work was supported by the Swedish Research Council for Health, Working Life, and Welfare [2018-00864 to C.N]; the Crafoord Foundation [20180659 to C.N] and the Magnus Bergvall Foundation [201802669 to C.N].

Conflicts of interests

None declared.

For peer review only - http://bmjopen.bmj.com/site/about/guidelines.xhtml

Ethics approval

The study was approved by the Swedish Ethical Review Authority (no. 2019-03138). Ethical approval to reapproach the responders with additional questionnaires has been obtained.

Patient and public involvement

The study population was the sex- and age-matched controls to the cases in the original studies, but otherwise constituted a representative sample of the Swedish general population in terms of sociodemographic characteristics. Members of the public participated in the pilot of the survey questions, and we implemented their feedback to improve the content of and the formulations in the questionnaire.

Data availability statement

The data underlying this article cannot be shared publicly due to the privacy of the individuals that participated in the study. Register data are obtained from third parties and are not publicly available. However, data from the TABOO cohort can be made available for research upon reasonable request, and after approval from the Swedish Ethical Review Authority.

Acknowledgements

The authors are grateful to the pilot participants that provided constructive input to improve the clarity and validity of the questionnaire. We also express a big thank you to all the study

For peer review only - http://bmjopen.bmj.com/site/about/guidelines.xhtml

participants that dedicated their time and effort to help us establish a unique research infrastructure that will move the field of health effects related to body modifications forward.

References

1. Piccinini P, Pakalin S, Contor L, Bianchi I, Senaldi C. Safety of tattoos and permanent make-up: Final report. EUR 27947. Luxembourg (Luxembourg): *Publications Office of the European Union*; 2016. JRC101601.

2. Klügl I, Hiller KA, Landthaler M, Bäumler W. Incidence of health problems associated with tattooed skin: a nation-wide survey in German-speaking countries. *Dermatology* 2010;**221**:43-50.

3. Manso M, Pessanha S, Guerra M, Reinholz U, Afonso C, Radtke M, Lourenço H, Carvalho ML, Buzanich AG. Assessment of toxic metals and hazardous substances in tattoo inks using Sy-XRF, AAS, and Raman spectroscopy. *Biol Trace Elem Res* 2019;**187**:596-601.

4. Wang X, Josefsson L, Meschnark S, Lind ML, Emmer Å, Goessler W, Hedberg YS. Analytical survey of tattoo inks - a chemical and legal perspective with focus on sensitizing substances. *Contact Dermat* 2021;**85**:340-53.

5. Swedish Medical Products Agency. Kontroll av tatueringsfärger för tatuering och permanent makeup med analysprotokoll - 2020. *Rapport från Läkemedelsverket* 2020. URL: https://www.lakemedelsverket.se/491725/globalassets/dokument/publikationer/tatueringsfarger/kontroll-av-tatueringsfarger-med-analysprotokoll-2020.pdf. Accessed 2022-10-26.

For peer review only - http://bmjopen.bmj.com/site/about/guidelines.xhtml

6. Vasold R, Naarmann N, Ulrich H, Fischer D, König B, Landthaler M, Bäumler W. Tattoo pigments are cleaved by laser light-the chemical analysis *in vitro* provide evidence for hazardous compounds. *Photochem Photobiol* 2004;**80**:185-90.

7. Engel E, Spannberger A, Vasold R, König B, Landthaler M, Bäumler W. Photochemical cleavage of a tattoo pigment by UVB radiation or natural sunlight. *J Dtsch Dermatol Ges* 2007;**5**:583-9.

8. Cui Y, Spann AP, Couch LH, Gopee NV, Evans FE, Churchwell MI, Williams LD, Doerge DR, Howard PC. Photodecomposition of Pigment Yellow 74, a pigment used in tattoo inks. *Photochem Photobiol* 2004;**80**:175-84.

9. IARC working group on the evaluation of carcinogenic risks to humans. Some non-heterocyclic polycyclic aromatic hydrocarbons and some related exposures. *IARC Monogr Eval Carcinog Risks Hum* 2010;**92**:1-853.

10. IARC working group on the evaluation of carcinogenic risks to humans. Carbon black, titanium dioxide, and talc. *IARC Monogr Eval Carcinog Risks Hum* 2010;**93**:1-413.

11. Lehner K, Santarelli F, Vasold R, Penning R, Sidoroff A, König B, Landthaler M, Bäumler W. Black tattoos entail substantial uptake of genotoxicpolycyclic aromatic hydrocarbons (PAH) in human skin and regional lymph nodes. *PloS one* 2014;**9**:e92787.

12. Schreiver I, Hesse B, Seim C, Castillo-Michel H, Villanova J, Laux P, Dreiack N, Penning R, Tucoulou R, Cotte M, Luch A. Synchrotron-based *ν*-XRF mapping and *µ*-FTIR microscopy enable to look into the fate and effects of tattoo pigments in human skin. *Sci Rep* 2017;**7**:11395.

13. Bäumler W. Absorption, distribution, metabolism and excretion of tattoo colorants and ingredients in mouse and man: the known and the unknown. *Curr Probl Dermatol* 2015;**48**:176-84.

For peer review only - http://bmjopen.bmj.com/site/about/guidelines.xhtml

14. Sepehri M, Sejersen T, Qvortrup K, Lerche CM, Serup J. Tattoo pigments are observed in the Kupffer cells of the liver indicating blood-borne distribution of tattoo ink. *Dermatology* 2017;**233**:86-93.

15. Ludvigsson JF, Otterblad-Olausson P, Pettersson BU, Ekbom A. The Swedish personal identity number: possibilities and pitfalls in healthcare and medical research. *Eur J Epidemiol* 2009;**24**:659-67.

16. Westerdahl J, Anderson H, Olsson H, Ingvar C. Reproducibility of a self-administered questionnaire for assessment of melanoma risk. *Int J Epidemiol* 1996;**25**:245-51.

17. Fitzpatrick TB. The validity and practicality of sun-reactive skin types I through VI. *Arch Dermatol* 1988;**124**:869-71.

18. Engdahl B. Kvalitetsdeklaration: Statistik om hjärtinfarkter 2020. *Socialstyrelsen* 2021. URL: https://www.socialstyrelsen.se/globalassets/sharepoint-dokument/artikelkatalog/statistik/2021-12-7648-kvalitetsdeklaration.pdf. Accessed 2022-10-26.

19. Lovering C. Kvalitetsdeklaration: statistik om stroke 2020. *Socialstyrelsen* 2021. URL: https://www.socialstyrelsen.se/globalassets/sharepoint-dokument/artikelkatalog/statistik/2021-12-7643-kvalitetsdeklaration.pdf. Accessed 2022-10-26.

20. Ohlin M, Otterdal P. Det statistiska registrets framställning och kvalitet: läkemedelsregistret. *Socialstyrelsen* 2021. URL: https://www.socialstyrelsen.se/globalassets/sharepoint-dokument/dokument-webb/statistik/statistiska-registrets-framstallning-kvalitet-lakemedelsregistret.pdf. Accessed 2022-10-26.

For peer review only

21. de Munter J. Det statistiska registrets framställning och kvalitet: dödsorsaksregistret. *Socialstyrelsen* 2022. URL: https://www.socialstyrelsen.se/globalassets/sharepoint-dokument/artikelkatalog/statistik/2022-5-7909.pdf. Accessed 2022-10-26.

For peer review only

For peer review only - http://bmjopen.bmj.com/site/about/guidelines.xhtml

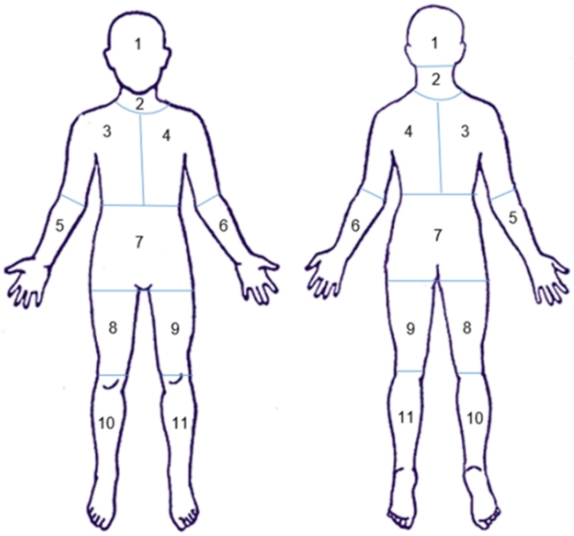

Figure 1. Body manikin used to retrieve information on anatomical site and approximate size of tattooed body surface.

855x481mm (38 x 38 DPI)

Supplementary file

Table S1. Detailed description of exposure data and personal characteristics collected through the TABOO questionnaire.

Variable	Who got the question	Response options	Multiple responses possible	Comment
Tattoos				
Tattoo status	All	Yes (including any removed tattoo)<br>No		
Type of tattoo	Tattoo status = yes	Decorative<br>Cosmetic<br>Medical	x	
Age at first tattoo	Tattoo status = yes	Years		
Age at last tattoo	Tattoo status = yes	Years		
Tattooed anatomical sites	Tattoo status = yes	1: Head<br>2: Neck<br>3: Right upper arm, chest, upper back<br>4: Left upper arm, chest, upper back<br>5: Right lower arm, hand<br>6: Left lower arm, hand<br>7: Abdomen, groin, genitals, lower back, buttocks<br>8: Right thigh, knee<br>9: Left thigh, knee<br>10: Right lower leg, foot<br>11: Left lower leg, foot	x	The numbered response options refer to anatomical sites in Figure 1, which was included in the questionnaire
Area of tattooed body surface	Tattoo status = yes	<1 hand<br>1 to 5 hands<br>>5 hands		
By whom and where tattoo was made	Tattoo status = yes	Professional tattoo artist in tattoo studio<br>Professional tattoo artist at other facility<br>Cosmetic tattoo artist in cosmetic tattoo studio, beauty salon, or plastic surgery clinic<br>Health care professional at hospital or other health care establishment	x	

For peer review only

		Other irrespective of where		
Number of tattoos	Tattoo status = yes	1<br>2 to 3<br>4 to 5<br>6 to 9<br>≥10		Tattoos considered separate if ≥20 cm between them<br>Cover-ups counted as 2 tattoos
Number of tattoo sessions	Tattoo status = yes	1<br>2 to 3<br>4 to 5<br>6 to 9<br>≥10		
Tattooed under the influence of alcohol or drugs	Tattoo status = yes	Yes<br>No		
Tattoo colours	Tattoo status = yes	Black<br>Gray<br>Brown<br>red<br>Blue<br>Green<br>Yellow<br>White<br>Purple<br>Pink<br>Orange<br>Turquoise<br>Skin tone (injected colour)<br>Other	x	
Geographical regions of tattooing	Tattoo status = yes	Sweden<br>Nordic countries (not Sweden)<br>Rest of Europe (not Nordic countries)<br>Asia<br>Oceania<br>USA<br>Other	x	
Other body modifications				
Piercing status	All	Yes<br>No, had earlier<br>No, never		Including earlobes
Scarification status	All	Yes<br>No		Scars obtained from branding or cutting
Henna tattoo status	All	Yes, black<br>Yes, red<br>No	x	Tattoo painted on skin
Laser treatment: Tattoo removal	Tattoo status = yes	Yes<br>No		
Laser treatment: Hair removal	All	Yes<br>No		Intense pulsed light (IPL given as an example)
Laser treatment: Mole or hyperpigmentation removal	All	Yes<br>No		Intense pulsed light (IPL given as an example)

For peer review only

Cohort Profile: The Swedish Tattoo and Body Modifications Cohort (TABOO)

Laser treatment: Thread vein removal	All	Yes<br>No		Intense pulsed light (IPL given as an example)
Laser treatment: Facial wrinkles reduction	All	Yes<br>No		Intense pulsed light (IPL given as an example)
Laser treatment: Other reason	All	Yes<br>No		Intense pulsed light (IPL given as an example)
Anatomical site of treatment	Laser treatment (specified) = yes	Face<br>Arms<br>Legs<br>Back<br>Chest and/or abdomen<br>Other	x	Repeated for each of the six reasons for laser treatment
Where laser treatment was performed	Laser treatment (specified) = yes	Beauty salon, beauty clinic, or plastic surgery clinic<br>Health care establishment<br>Home<br>Other<br>(For tattoo removal, the option tattoo artist was provided)		Repeated for each of the six reasons for laser treatment
Number of laser treatments	Laser treatment (specified) = yes	1 to 2<br>3 to 4<br>5 to 6<br>7 to 8<br>≥9		Repeated for each of the six reasons for laser treatment
Hair dyeing	All	Yes<br>No	x	Included dyeing, toning, and bleaching
Frequency last 5 years	Hair dyeing status= yes	≥3 times per year<br>1 to 2 times per year<br><1 time per year		
Where hair dyeing was performed	Hair dyeing status = yes	Hair salon<br>Home<br>Other	x	
Age at first hair dyeing	Hair dyeing status = yes	<18 years<br>18 to 29 years<br>≥29 years		
Pigmentation and sun habits				
Childhood eczema	All	Yes<br>No<br>Don't know		
Atopic childhood eczema	All	Yes<br>No<br>Don't know		Atopy defined as dry and itchy skin, often with rash, bumps, and sometimes with oozing blisters

For peer review only - http://bmjopen.bmj.com/site/about/guidelines.xhtml

Cohort Profile: The Swedish Tattoo and Body Modifications Cohort (TABOO)

Natural eye colour	All	Blue<br>Gray<br>Green<br>Yellowish<br>Light brown<br>Dark brown<br>Mottled/mixed<br>Heterochromia	
Natural skin tone[1]	All	Very fair<br>Fair<br>Medium<br>Olive, light brown<br>Brown<br>Dark brown	Question with illustrated examples
Freckles after sun exposure	All	Yes, many<br>Yes, few<br>No	
Skin reaction to first sun exposure	All	Never burns but tans darker<br>Burns slightly then tans<br>Burns with pain lasting ≤1 day<br>Burns with pain lasting ≥2 days (may blister)	
Severe sunburn, <13 years	All	Yes, multiple occasions<br>Yes, single occasion<br>No	
Severe sunburn, >13 years	All	Yes, multiple occasions<br>Yes, single occasion<br>No	
Recreational sun travel, <13 years	All	Yes, yearly<br>Yes, but not regularly<br>No	
Recreational sun travel, >13 years	All	Yes, yearly<br>Yes, but not regularly<br>No	
Sunscreen use in childhood	All	Yes, always<br>Yes, sometimes<br>No<br>Don't know	
Sunscreen use in adulthood	All	Yes, always<br>Yes, sometimes<br>No<br>Don't know	
Sunbed use, <13 years	All	Yes, multiple occasions	

For peer review only

Cohort Profile: The Swedish Tattoo and Body Modifications Cohort (TABOO)

		Yes, single occasion<br>No	
Sunbed use, >13 years	All	Yes, multiple occasions<br>Yes, single occasion<br>No	
Occupational sun exposure (April-September)	All	Yes<br>No<br>Not working	
Duration of occupational sun exposure (h per week)	Occupational sun exposure = yes	≤5<br>6 to 20<br>>20	
Changed sun habits after being tattooed	Tattoo status = yes	Yes, sunbath more<br>Yes, sunbath less<br>Yes, same extent of sunbathing but cover or use sunscreen on tattooed skin<br>No	
Tobacco smoking	All	Yes, daily<br>Yes, but not daily<br>No, but previously<br>No, never	
Amount smoked per day	Tobacco smoking = yes	Cigarettes<br>E-cigarettes<br>Cigars<br>Cigarillos<br>Pipes	Numbers of each type
Age when started smoking	Tobacco smoking = yes	Years	
If quit smoking, age at quitting	Tobacco smoking = yes	Years	
Snuff use	All	Yes, daily<br>Yes, but not daily<br>No, but previously<br>No, never	Only nicotine-containing snuff
Portions per day	Snuff use = yes	Number	
Age when started snuff use	Snuff use = yes	Years	
If quit snuff use, age at quitting	Snuff use = yes	Years	
Alcohol consumption frequency	All	Daily<br>4-6 days per week<br>2-3 days per week<br>1 day per week<br>2-3 days per month<br>Rare occasions<br>Never	Beverages with ≥3.5 % alcohol

[1]According to the Fitzpatrick Skin Phototype Classification[17].

For peer review only

For peer review only - http://bmjopen.bmj.com/site/about/guidelines.xhtml

Cohort Profile: The Swedish Tattoo and Body Modifications Cohort (TABOO)

Table S2. Diagnostic codes according to the International Classification of Diseases (ICD), versions 10 and 9, used to retrieve outcome data from the National Patient Register in 2021.

Version 10		Version 9	
ICD-10	Level of detail[1]	ICD-9	Level of detail
D86	Full	135	Full
E03-06	Full	240.0	Full
F32	Category	240.9	Full
F41	Category	241.0-1	Full
F43	Category	241.9	Full
F45.4	Full	242.0-4	Full
G43-44	Category	242.8-9	Full
G53.2	Full	243	Full
H22.1	Full	244.3	Full
I41.8	Full	244.8-9	Full
M05.9	Full	245.0-4	Full
M06.0	Full	245.8-9	Full
M06.9	Full	246.1-2	Full
M25.5	Full	246.8	Full
M63.3	Full	296.1	Category
M79.1	Full	296.9	Category
M79.6-7	Full	298.0	Category
M54	Category	300.0-4	Category
R10	Category	306.0	Full
R51	Category	307.8	Full
R52.1-2	Full	308.0-4	Category
R52.9	Full	308.9	Category
		309.0-4	Category
		309.8-9	Category
		311	Category
		346.0-2	Category
		346.8-9	Category
		352.6	Full
		364.1A	Full
		364.8	Full
		429.0	Full
		536.8	Category
		574.2	Category
		714.0	Full
		714.8	Full
		719.4	Full
		723.1	Category
		723.4	Category
		723.6	Category
		724.4	Category
		724.1-3	Category
		724.5	Category
		724.8-9	Category
		729.1-2	Full
		729.5	Full
		780.9	Full
		784.0	Category
		789.0	Category

[1]Level of detail refers to the amount of information pulled from the ICD alphanumeric code. Full is the complete set of characters, category is the first three positions.

For peer review only - http://bmjopen.bmj.com/site/about/guidelines.xhtml

Cohort Profile: The Swedish Tattoo and Body Modifications Cohort (TABOO)

Table S3. Anatomical Therapeutic Chemical (ATC) codes used to retrieve outcome data from the National Prescribed Drug Register in 2021.

ATC-code	Level of detail[1]
A05AA02	Chemical substance
D0	Chemical subgroup
D11AH	Chemical subgroup
H02-3	Chemical substance
J01	Pharmacological subgroup
J06BA	Chemical substance
L	Chemical substance
M01-2	Chemical substance
N02	Chemical subgroup
N05-6	Pharmacological subgroup
N07	Chemical subgroup
R03	Chemical subgroup
R06	Pharmacological subgroup
S01B, C, and G	Chemical subgroup
S03B, and C	Chemical subgroup

[1]Level of detail refers to the amount of information pulled from the ATC alphanumeric code.

For peer review only - http://bmjopen.bmj.com/site/about/guidelines.xhtml
